# Cyclic FEE Peptide Improves Human Sperm Movement Parameters without Modification of Their Energy Metabolism

**DOI:** 10.3390/ijms222011263

**Published:** 2021-10-19

**Authors:** Nathalie Le Foll, Jean-Christophe Pont, Audrey L’Hostis, Thomas Guilbert, Frédéric Bouillaud, Jean-Philippe Wolf, Ahmed Ziyyat

**Affiliations:** 1 Institut Cochin, Université de Paris, INSERM, CNRS, 75014 Paris, France; nathalielefoll@yahoo.fr (N.L.F.); jchristophe.pont@gmail.com (J.-C.P.); audrey.l-hostis@frema-pharma.com (A.L.); thomas.guilbert@inserm.fr (T.G.); frederic.bouillaud@inserm.fr (F.B.); ahmed.ziyyat@u-paris.fr (A.Z.); 2 Service D’histologie, D’embryologie, Biologie de la Reproduction, AP-HP, Hôpital Cochin, 75014 Paris, France

**Keywords:** fertilization, ATP, spermatozoa, motility

## Abstract

Cyclic fertilin peptide (cFEE: phenylalanine, glutamic acid; glutamic acid) improves gamete interaction in humans. We investigate whether it could be via improvement of sperm movement parameters and their mitochondrial ATP production. Sperm movement parameters were studied using computer-assisted sperm analysis (CASA) in sperm samples from 38 patients with normal sperm in medium supplemented with cyclic fertilin against a control group. Sperm mitochondrial functions were studied using donor’s sperm, incubated or not with cFEE. It was evaluated by the measurement of their ATP production using bioluminescence, their respiration by high resolution oxygraphy, and of mitochondrial membrane potential (MMP) using potentiometric dyes and flow cytometry. cFEE significantly improved sperm movement parameters and percentage of hyperactivated sperm. Impact of inhibitors showed OXPHOS as the predominant energy source for sperm movement. However, cFEE had no significant impact on any of the analyzed mitochondrial bioenergetic parameters, suggesting that it could act via a more efficient use of its energy resources.

## 1. Introduction

The α6β1 integrin has been suggested to be the receptor for fertilin β in mice, based on the inhibition of fertilin β binding to oocyte, induced by specific monoclonal antibodies [1,2,3]. Anti-integrin α6 antibody inhibited both fertilization rate and fertilization index of cumulus-intact or zona-free mouse assays. These inhibitions mostly occurred when sperm were pre-incubated with the antibody, suggesting that this effect could be mediated by sperm. Indeed, the anti-integrin α6 antibody has little effect on oocyte fertilization ability [4].

The cyclic peptide FEE (cFEE) reproduces the binding site of the disintegrin domain of the human fertilin β (ADAM2) [5]. This molecule is highly conserved among mammalian species, but has some species specificities. While the cFEE peptide (phenylalanine, glutamic acid; glutamic acid) is active in human, mouse gametes require the cQDE peptide (glutamine; aspartic acid; glutamic acid). In mouse, we have previously shown that cQDE significantly increases the IVF fertilization rate [6]. Sperm from mice lacking fertilin β were shown to be deficient in sperm–egg membrane adhesion, sperm–egg fusion, migration from the uterus into the oviduct, and binding to the egg zona pellucida [7].

In human, using zona-free unfertilized oocytes donated by patients according the French bioethics laws, we showed that cFEE increases the fertilization index by 90% [8]. We therefore investigated the impact of cFEE on sperm functions.

In the present work, we studied all the possible effects of the cFEE peptide on the sperm functions that are involved in sperm fertilizing ability including sperm vitality, motility, and acrosome reaction. We also investigated its possible side effects on sperm quality and explored DNA fragmentation.

Indeed, the motility of sperm is a prerequisite for its fertilizing ability. The movement results from the beating of flagella, which relies upon the conversion of ATP chemical energy into mechanical work by the dynein ATPase. ATP is also the substrate for adenylyl cyclase producing cyclic AMP, which also stimulates sperm movement. The source of ATP is still a matter of debate. Ruiz-Pesini et al. [9] reported that oxidative phosphorylation (OXPHOS) is the main source of energy in the spermatozoa. Others such as William and Ford [10] concluded that the glycolytic ATP concentration is required for vigorous motility and hyperactivation in human sperm. In fact, many parameters may influence the metabolism of ATP production: oxygen concentration, media composition, and temperature. Presence and concentration of inhibitors also have to be considered and have varied a lot between reports, thus rendering their comparison difficult. Glycolysis takes place in the fibrous sheath of the principal piece of mammalian sperm flagella [11,12], while OXPHOS including the mitochondrial electron transport chain and the ATP synthase is located in the sperm mitochondrial sheath in the sperm mid piece. The electron transport chain consists of respiratory complexes I to IV. It transports electrons from donors such as NADH and FADH to molecular oxygen and pumps out protons across the inner mitochondrial membrane. This process generates the inner mitochondrial membrane potential (MMP), which is the source of energy for ATP production by the complex V (also called ATP synthase).

To assess the potential role of cFEE on ATP production, we analyzed the three main aspects of glycolysis and OXPHOS: electron transport (disclosed by oxygen consumption), MMP, and ATP synthesis. This comprehensive approach of spermatozoa bioenergetics showed that cFEE does not significantly modify ATP production by OXPHOS or glycolysis. However, we showed that cFEE improves sperm movement parameters and increases by 30% the proportion of spermatozoa that are hyperactivated without affecting neither sperm vitality, DNA quality, nor acrosome reaction. These results suggest that the positive cFEE impact on sperm movement might be born out of a more efficient use of ATP turnover.

## 2. Results

### 2.1. cFEE Binds to Spermatozoa

**Figure 1:**
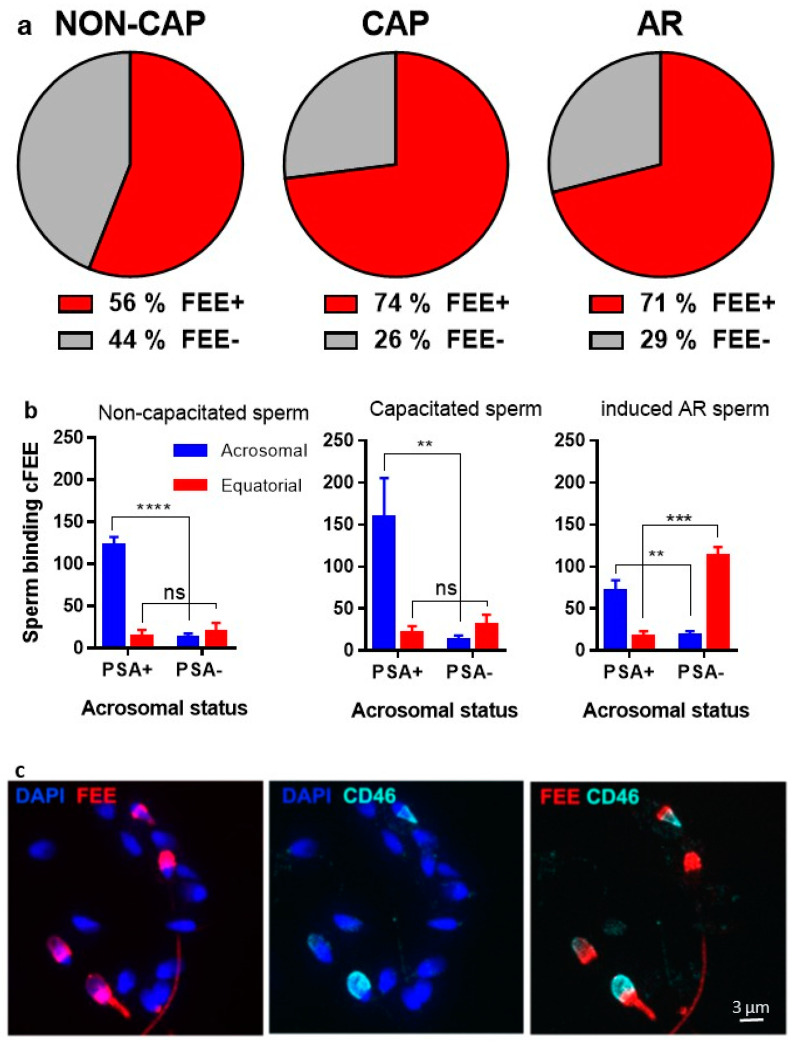

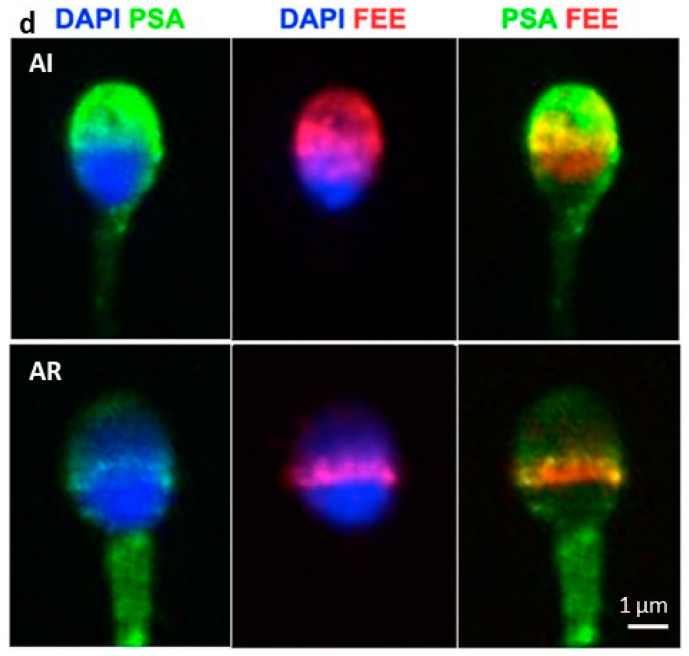
cFEE binding to sperm. cFEE binding pattern on human sperm depends on acrosome status. (**a**) Percentage of human sperm binding cFEE in fresh semen sample (NON-CAP), after capacitation (CAP) and after acrosome-reaction (AR). Dunn’s multiple comparisons after ANOVA test showed no significant differences between the groups. (**b**) Localization of cFEE binding to human sperm in NON-CAP, CAP, and induced AR conditions. cFEE binding localization is shown as sperm number (mean ± s.e.m) for NON-CAP sperm (**left**), CAP sperm (**middle**), and induced AR (**right**). A two-way ANOVA and Sidak’s multiple comparisons test showed a significant difference in cFEE acrosomal localization between the positive and the negative PSA groups independently of sperm status (NON-CAP, **** *p* < 0.0001; CAP, ** *p* = 0.005, iAR, ** *p* = 0.009 and *** *p* = 0.0002) and in cFEE equatorial localization only in the iAR group, in which the percentage of acrosome-reacted sperm is important (*p* = 0.0002). These data were obtained from three independent experiments where we counted over 300 sperm in each of the subgroups (NON-CAP, CAP, and AR) in each experiment. (**c**) FEE and CD46 immunofluorescence on capacitated human sperm. Nuclei were counterstained by DAPI. (**d**) PSA and FEE immunofluorescence on capacitated human sperm. The upper panel shows capacitated acrosome-intact (AI) spermatozoon, which binds cFEE on the acrosome. The bottom panel shows capacitated acrosome-reacted (AR) spermatozoon, which binds FEE on the equatorial region. Nuclei were counterstained by DAPI. NON-CAP: non-capacitated, CAP: capacitated, AI: acrosome-intact, AR: acrosome-reacted.

We showed in a previous study that cFEE increases the fusion index of human gametes [8], but the underlying mechanism of cFEE action is unknown. In the present study, we first assessed whether cFEE is able to bind human spermatozoa. In order to check the binding of cFEE to sperm and the localization of its potential receptor according to the sperm maturation status, we incubated the peptide with non-capacitated, capacitated, and induced acrosome-reaction sperm. We observed that cFEE binds to 56% of non-capacitated, 74% of capacitated, and 71% of induced acrosome-reaction sperm (Figure 1a). We then analyzed the localization of cFEE binding to sperm as protein dynamics on sperm head play a crucial role in the path to sperm fertilizing ability along with capacitation and acrosome reaction [13,14]. The majority of the non-capacitated and capacitated acrosome-intact sperm (cFEE + PSA+) showed a cFEE binding restricted to the acrosome (Figure 1b,d, upper panels). However, induced acrosome-reacted sperm (cFEE and PSA-negative or cFEE and CD46 positive) showed a shift toward equatorial cFEE binding (Figure 1b,d, bottom panels; Figure 1c). Thus, cFEE localization on human sperm seems to be mostly dependent on acrosomal status.

### 2.2. cFEE Potentiates Sperm Movement without Toxic Impact

#### 2.2.1. cFEE Increases Sperm Movement Parameters

Since cFEE improves the fusion index in human gametes, we investigated whether the peptide modifies sperm functions including sperm movement parameters. Results for scramble and cFEE treated sperm using CASA analysis are shown in Table 1 (*n* = 38 sperm). Several parameters including average path velocity (VAP), curvilinear velocity (VCL), and straight path velocity (VSL) were significantly increased in the cFEE group when compared to the scramble group (control). BCF, STR, and LIN remained unchanged. The proportion of hyperactivated spermatozoa was significantly increased by 29% in the cFEE compared to the scramble group (13.6% versus 10.5%, *p* = 0.002) according to the criteria of Mortimer and Swan [15].

#### 2.2.2. cFEE Does Not Affect Sperm Vitality

The sperm vitality was assessed using flow cytometry analysis after propidium iodide staining. Comparison of mean values and standard deviation of sperm vitality after selection in the presence of cFEE, the scramble peptide, and control culture medium are reported in Table 2. The Wilcoxon test analysis showed no significant differences. Sperm vitality was not impaired by either the cyclic peptide at a concentration up to 200 µM or by the scramble peptide at 200 µM.

#### 2.2.3. cFEE Does Not Induce Acrosome Reaction

As acrosome reaction is a required step for fertilization, we assessed the effect of cFEE on AR induction. The proportion of acrosome reacted sperm was measured by flow cytometry after acrosome markers staining. After 18 h of incubation with cFEE or scramble peptide, the percentages of acrosome reacted sperm were similar, 16.8 ± 1.3% and 16.6 ± 1.3% (*n* = 8), respectively. Following the calcium ionophore induced acrosome reaction, the percentage of acrosome reacted sperm in cFEE or scramble peptide groups were 49.2 ± 20.1% and 48.2 ± 20.0%, (*n* = 8), respectively. No significant differences were observed, suggesting that cFEE modifies neither the spontaneous nor the induced acrosome reaction. As the spermatozoa activation is a prerequisite for the acrosome reaction to occur, we concluded that cFEE does not change the status of sperm capacitation either.

#### 2.2.4. cFEE Does Not Alter Nuclear Quality

The degree of sperm nucleus fragmentation is a reliable indicator of reproductive success. Evaluation of DNA fragmentation was made on 10 sperm samples. When sperm samples were maintained for 3 h in the presence of 100 µM cFEE, 3.4% of living spermatozoa exhibited DNA fragmentation, a level similar to that of control spermatozoa (3.2%; *p* = 0.6). When they were maintained overnight in the same incubation medium, the total DNA fragmentation switched to 21.4% and 26.1% (*p* = 0.01) respectively. Interestingly, the level of living sperm DNA fragmentation was not modified, 8.6% versus 6.8% of the live control spermatozoa (*p* = 0.06).

### 2.3. Impact of cFEE on Energy Production

Before analyzing the impact of cFEE on energy production, we analyzed the Ferticult composition (the medium used to assess the cFEE effects on sperm movement) and found that it contains substrates for both glycolysis and OXPHOS (Supplementary Materials S1). Next, we analyzed the energetic requirement for sperm movement in basal conditions and after glycolysis or OXPHOS inhibitions (see Supplementary Materials, Table S1 and Figure S1). We quantified the respective part of glycolysis and OXPHOS in the ATP production (Figure S2).

#### 2.3.1. ATP Measurement

The significant impact of cFEE on sperm motility led us to address its impact on energy production. We first analyzed the impact of a 3-h incubation with or without cFEE on the ATP steady state, before and after inhibition of either OXPHOS or glycolysis as above. Data obtained from four independent sperm from healthy donors, measured without cFEE (*n* = 46 samples, 469 ± 51 pmol/million sperm) or in the presence of 100 µM cFEE (*n* = 48, 457 ± 43 pmol/million sperm), led us to conclude that cFEE had no impact on the ATP steady state (*p* = 0.26).

**Figure 2:**
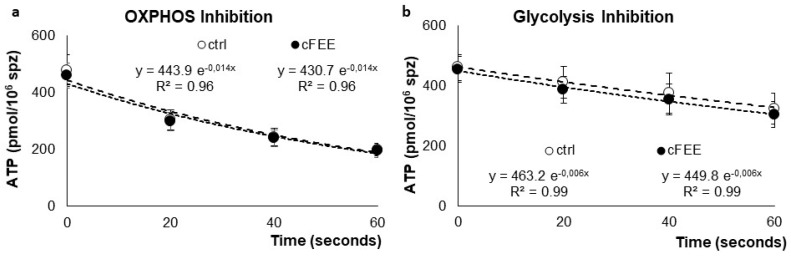
Average ATP concentrations in pmol/million sperm at different times after inhibition of (**a**) OXPHOS by oligomycin and (**b**) glycolysis by iodoacetate in donor sperm previously incubated in control medium (white circles) or in medium supplemented with cFEE (black circles).

The slopes of ATP consumption, after OXPHOS inhibition by oligomycin (Figure 2a) or glycolysis inhibition by iodoacetate (Figure 2b), were similar. Their values, calculated with the exponential regression curves after OXPHOS inhibition, were 360 and 349 pmol ATP consumed per minute for sperm incubated without and with 100 µM cFEE, respectively. After inhibition of the glycolysis pathway by iodoacetate, they were 164 and 160 pmol of ATP consumed per minute for sperm incubated without and with 100 µM cFEE, respectively. This difference was not statistically significant.

#### 2.3.2. Respiration Analysis

**Figure 3:**
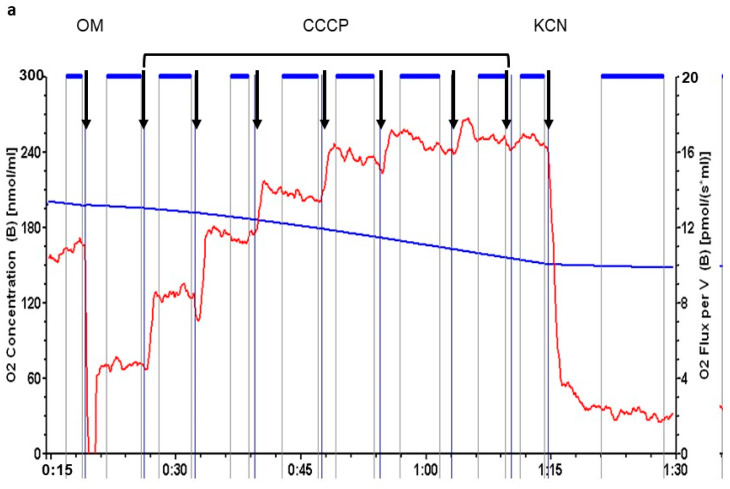

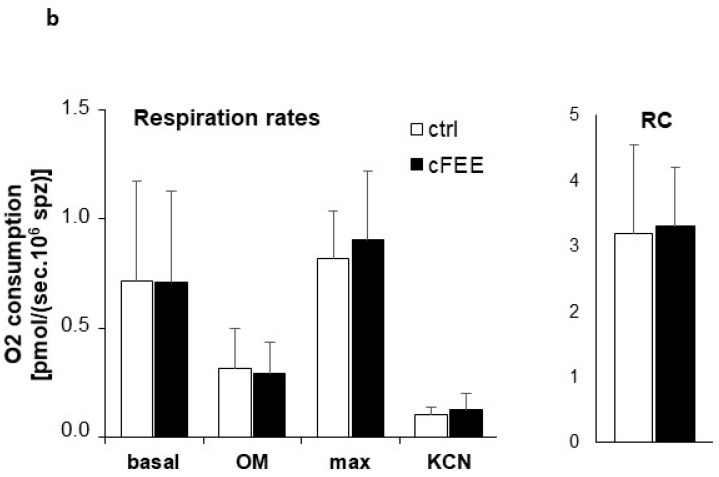
cFEE impact on sperm respiration. (**a**) Representative respiration analysis using Oroboros instrument; sperm concentration was 17.5 × 10^6^ spermatozoa/mL; black arrows = successive additions into Ferticult; OM = 1 µM oligomycin; CCCP = 1.25 µM CCCP; KCN = 1 mM KCN; horizontal blue lines between arrows represent periods chosen for calculation of the rate of oxygen consumption; blue curve with values in nmol per milliliter in the left axis = oxygen concentration in the medium; red curve with values in pmol per second and milliliter in the right axis = rate of oxygen consumption. (**b**) cFEE had no impact on sperm respiration parameters; ctrl = 7 independent sperm preparations from healthy donors after 3-h incubation without cFEE; cFEE = same samples but incubated in the presence of 100 µM cFEE; respiration rates are expressed as pmol O_2_ consumed per second and million sperm (pmol/(sec.10^6^ sperm)); Basal = respiration in basal conditions; OM = respiration after the addition of oligomycin, an inhibitor of ATP synthase, measuring the respiration rate not producing ATP; max = maximal respiration level obtained after successive additions of 1.25 µM CCCP, a protonophore dissipating the inner mitochondrial membrane potential; KCN = oxygen consumption after addition of potassium cyanide, an inhibitor of respiratory complex IV, measuring the non-respiratory oxygen consumption; RC = respiratory control defined as the ratio between basal respiration and respiration after oligomycin addition. Analysis used the Student *t* test to compare groups with or without cFEE and no difference was observed. Comparing each cFEE group to its direct control without cFEE using ANOVA, we obtained the same results.

Analysis of sperm respiration was performed using high resolution respirometry (Figure 3). During each respiration run, we evaluated four rates of oxygen consumption and calculated two parameters, respiratory control and respiratory reserve, based on the previously established rates. Initial respiration rate was measured in Ferticult (basal respiration). Inhibition of ATP synthase by 1 µM oligomycin (OM) then allowed for the evaluation of the residual oxygen respiration. It is the oxygen consumption that occurs while OXPHOS is inhibited and does not produce any ATP. Successive doses of 1.25 µM CCCP, a protonophore dissipating the inner membrane potential, were then added. It stimulates the mitochondrial respiration until reaching a maximal level. Finally, the addition of 1 mM potassium cyanide (KCN), an inhibitor of respiratory complex IV, evaluated the non-respiratory oxygen consumption, which was subtracted from the previous rates to obtain real respiratory rates.

The basal respiration rate of sperm is very low, less than 1 pmol oxygen consumed per second and million spermatozoa. Despite the high sensibility of the respirometer, we had to introduce very high numbers of spermatozoa to reach reliable respiration detection. We therefore pooled sperm from two donors for each measurement. Respiratory control, evaluated by the ratio between basal respiratory rate and rate under oligomycin inhibition, showed relatively efficient coupling in sperm. Respiratory reserve, defined as the difference between the maximal and basal respiration rates, remained modest at less than 50% of basal rate. All respiration parameters were similar in sperm incubated with cFEE and without.

#### 2.3.3. Evaluation of Mitochondrial Membrane Potential (MMP)

**Figure 4:**
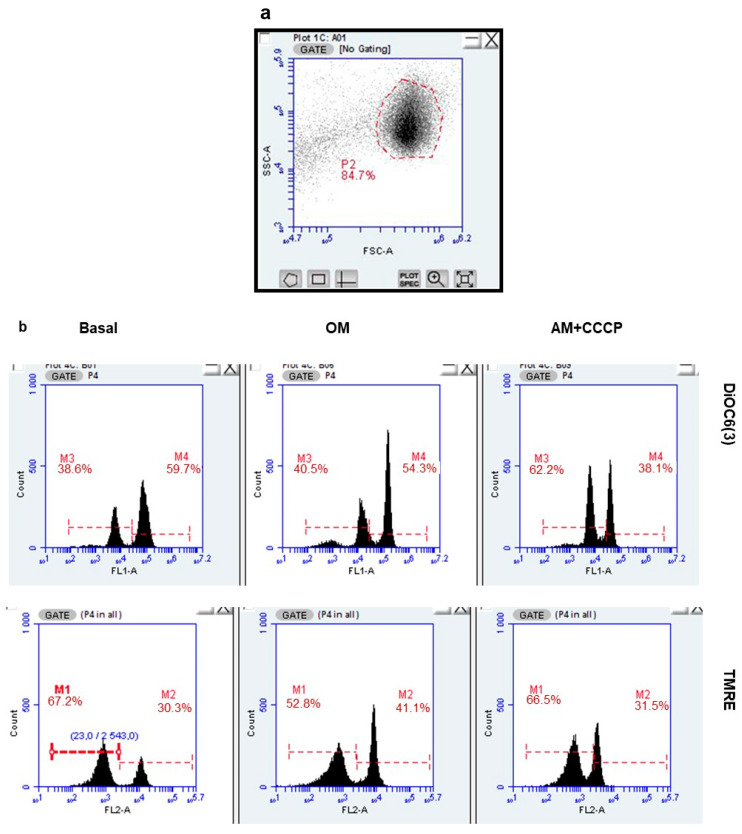

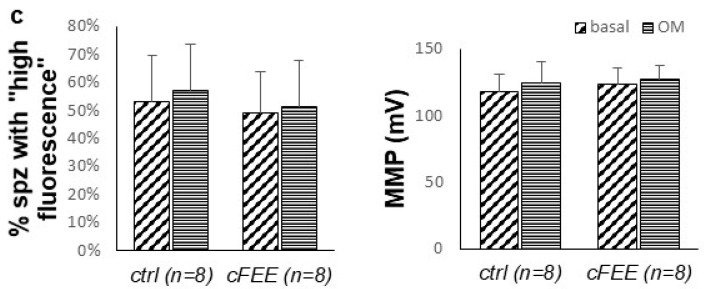
cFEE impact on mitochondrial membrane potential as measured by flow cytometry. (**a**). Spermatozoa were easily gated based on the size (FSC = forward scatter) and granularity (SSC = side scatter). (**b**) Two populations differing by their fluorescence signal were observed with both DiOC6(3) and TMRE probes, only the population with high fluorescence responded to the modulation of MMP, either increasing its signal upon ATP synthase inhibition by oligomycin (OM) or decreasing it upon MMP dissipation by the association of antimycin and CCCP (AM + CCCP). (**c**) cFEE had no impact on the proportion of spermatozoa with high fluorescence nor on their MMP calculated with the Nernst equation.

We analyzed the impact of cFEE on MMP using potentiometric probes whose fluorescence was analyzed by flow cytometry. Treatment with the association of 2.5 µg/mL antimycin A, a respiratory complex III inhibitor, and CCCP, a protonophore uncoupling agent, induces the complete dissipation of the MMP. We measured MMP in basal condition, in Ferticult medium, and after ATP synthase inhibition with 1 µM oligomycin. ATP synthase, being the main process using the proton gradient, oligomycin treatment should induce a maximal MMP signal. The spermatozoa could easily be gated based on their size and granularity (Figure 4a). Using TMRE or DiOC6(3) probes, the MMP values were distributed in two clearly separated sperm populations, roughly equal in size (Figure 4b). This was true in the basal condition and with oligomycin inhibition, and was more interestingly also true when inhibition was performed using simultaneously antimycin and CCCP. The population with low fluorescence had, in fact, a fluorescence signal lower than the signal obtained after complete MMP dissipation in the population with high fluorescence. This observation suggests that the fluorescent probe has either not entered inside the sperm mitochondria or has efficiently been exported out from the sperm population with low fluorescence.

We calculated the proportion of “fluorescent sperm” and their MMP in the basal condition and under inhibition by oligomycin, in eight independent sperm preparations from healthy donors. This was conducted after a 3-h incubation with and without 100 µM cFEE (Figure 4c). The MMP under oligomycin was significantly higher than that in the basal condition (125 mV versus 120 mV, *p* = 0.005 with the Wilcoxon signed Rank test, since data did not have a normal distribution). We did not observe any cFEE impact on any of these parameters (comparison with Mann–Whitney test).

**Figure 5:**
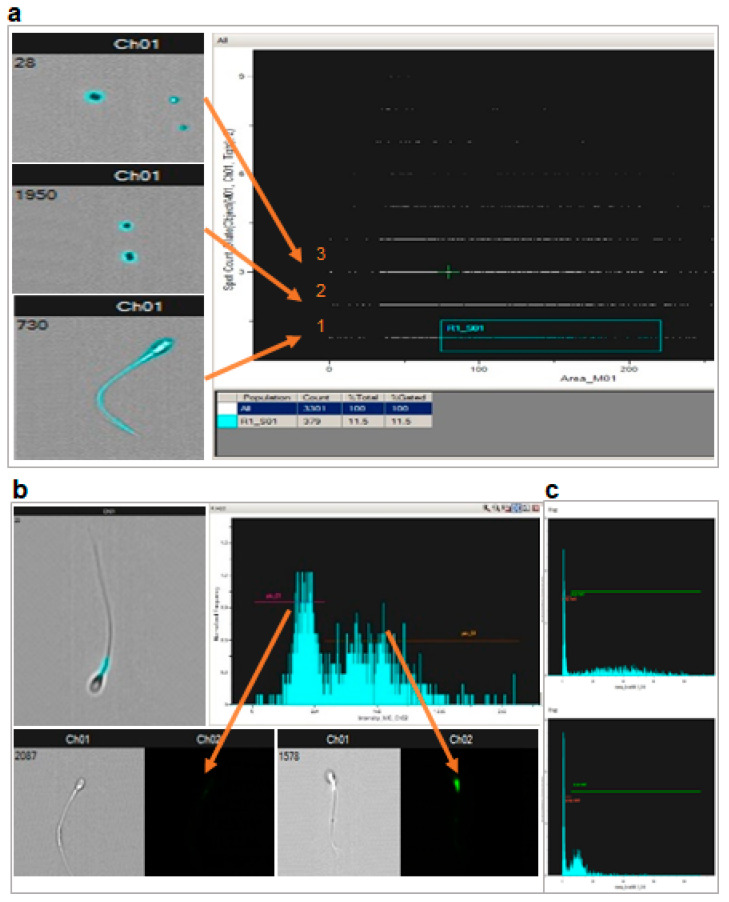

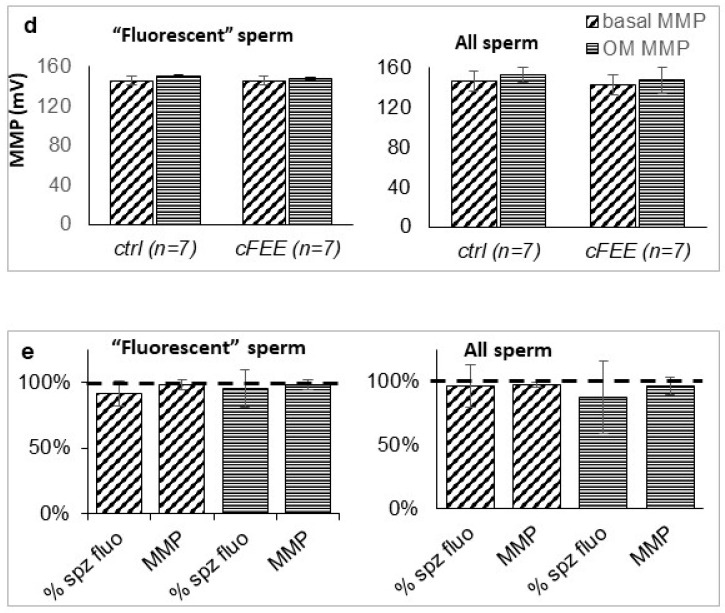
Flow cytometry coupled with microscopy confirmed the presence of sperm unstained by potentiometric fluorescence probes as well as the lack of impact on MMP of cFEE. (**a**) Scheme of the selection of relevant objects (arrows, 1: spermatozoa entirely fluorescent, 2 and 3: beads and debris) based on bright field images, using both the number per field and their size. (**b**) Non fluorescent objects were morphologically normal spermatozoa; fluorescent signal was localized in the middle piece of sperm as expected from a mitochondrial signal; the repartition of the fluorescence intensity allows for defining a population with very low fluorescence signal in a narrow “negative peak” (left arrow) and the rest of the population in a broad “positive peak” (right arrow). (**c**) The positive and negative peaks previously defined in the basal state were transferred to the histogram obtained with oligomycin or antimycin + CCCP inhibition, allowing for the evaluation of the proportion of fluorescent sperm (# sperm in positive peak/total # sperm analyzed) and MMP (according to the Nernst law taking as zero signals the fluorescence value in antimycin + CCCP). (**d**) cFEE had no impact on the MMP in basal conditions (obliquely shaded bars) or after oligomycin inhibition (horizontally hatched bars), whether analyzed only in the positive peak (“fluorescent” sperm) or in the whole sperm population (All sperm); MMP expressed in mV using the Nernst equation. (**e**) cFEE had no impact on the proportion of sperm in the positive peak (% fluorescent sperm) nor on the MMP in basal conditions (obliquely shaded bars) or after oligomycin inhibition (horizontally hatched bars) whether analyzed only in the positive peak (“fluorescent” sperm) or in the whole sperm population (All sperm); MMP and proportion of sperm in the positive peak expressed as % of the condition without cFEE in the 3-h incubation prior to analysis.

To address the nature of the “low MMP” population, we used flow cytometry coupled with fluorescence imaging contrast (ImageStreamX MKII- AMNIS Corp., Seattle, WA, USA) and staining with 100 nM TMRE (Figure 5). The method uses the initial selection of the objects of interest based on their morphology in bright field. In our case, successive use of algorithms, tight function mask then twice dilated binary filter, allowed for the recognition of all objects (sperm, smaller debris, calibration beads…) that appear highlighted by blue masks (Figure 5a). Ordering these objects by size and number per field allows the selection of the objects that are both lonely with a surface above 75 pixels. Visual inspection confirmed that the selected population contained only spermatozoa. They represented roughly 10% of the initial objects. The fluorescence signal of the sperm population was then analyzed as in regular cytometry, but with the possibility to individually visualize each object whose fluorescence was recorded (Figure 5b). The proportion of spermatozoa with very low fluorescence was roughly 50%; this was similar after sperm selection using a double (45–90%) or a triple Pursperm gradient (45–65–90%) procedure. Transfer to the two control situations (oligomycin or antimycin + CCCP inhibition) of the positive and negative peaks, defined in the basal state, allowed for calculating the proportion of fluorescent sperm (# sperm in positive peak/total # sperm analyzed) and MMP (according to Nernst law, taking as zero signals the fluorescence value under antimycin + CCCP inhibition).

With this technique, we analyzed seven independent sperm preparations after a 3-h incubation with or without cFEE. Although the MMP under oligomycin inhibition was globally higher than in the basal state (*p* = 0.003 with the paired *t* Student test on the 28 pairs of data), this difference did not reach significance in the smaller groups of seven pairs of data (Figure 5d). cFEE had no significant impact on the MMP nor on the proportion of the sperm belonging to the positive peak, either in the basal state or under oligomycin inhibition (Figure 5e).

## 3. Discussion

We have previously shown in mice that the cQDE peptide, equivalent to cFEE in human, increases the fertilization rate in the intact-cumulus assay. The cFEE peptide also increases the fertilization index using zona-free human oocytes. In fact, it increases sperm movement parameters and the percentage of human hyperactivated sperm by 30%, which should increase IVF fertilization rates. Since the sperm is moving faster, one can expect that it has an improved ATP production, which was not the case. Finally, the peptide had no deleterious effect on the nuclear quality: sperm DNA fragmentation was not modified after 24 h of incubation with the peptide, suggesting a good tolerance of the molecule by gametes.

We first showed that cFEE binds to sperm regardless of its maturation status (non-capacitated, capacitated, or acrosome reacted). Then, we sought to specify its location according to the maturation of the sperm. Indeed, essential sperm proteins (such as IZUMO1 or SPACA6), but also those that participated in the gamete interaction (such as integrins) relocated after the acrosomal reaction. This relocation itself seems necessary for fertilization. In mice, it has been shown that in the absence of the TSSK6 kinase, the relocation of IZUMO1 during the acrosomal reaction, from the acrosomal cap region to the equatorial segment, is not undertaken and sperm are then unable to fertilize [14]. The switch of cFEE from the acrosomal region to the equatorial segment indicates that its receptor on the sperm follows the same relocation as that of IZUMO1, suggesting that this could also be important for fertilization. Since the cFEE reproduces the binding site of the disintegrin domain of the human ADAM2 [5] and the disintegrin-like domain of ADAM2 was shown to bind to the α6β1 integrin on the egg plasma membrane [2], we can hypothesize an interaction between the peptide and this integrin. In addition, anti-integrin antibodies inhibit mouse in vitro fertilization and integrins also seem to relocate following AR [4,16]. Even if the sperm beta1 conditional deletion does not prevent male fertility, their sperm fertilize significantly less in vitro, suggesting both the participation of this integrin subunit in fertilization and the possible redundancy explaining in vivo fertility [17]. The cFEE could therefore bind to one or more integrins (or even to other receptors) and activate a signaling pathway favorable to fertilization.

The other aspect likely to explain the improvement in fertilization is that of the increase in sperm hyperactivation in the presence of cFEE. Additionally, the modulation of the energy production mechanism underlying the impact on sperm movement is an attractive hypothesis since its movement directly depends upon ATP production. The metabolic pathways of ATP production in sperm are still debated: depending on the available substrates and the physico-chemical conditions in which they participate, different metabolic pathways may be activated. We analyzed the source of ATP production in Ferticult (Supplementary Materials S1), with a substantial amount of both glycolysis and OXPHOS substrates, and in optimal incubation conditions at 37 °C with 5% CO_2_. In these conditions, ATP is produced by both metabolic pathways, predominantly by OXPHOS, which produced roughly 60% of total ATP. Inhibition experiments showed that sperm movement depended on both pathways, albeit again mostly on OXPHOS in our experimental conditions (Supplementary Materials Table S1 and Figure S1). The apparent discrepancy between immediate sperm movement arrest and progressive decline in ATP upon OXPHOS inhibition suggests that a threshold of ATP production may exist under which the sperm will no longer be able to move. Furthermore, sperm movement most likely stops before complete ATP depletion because the physiological regulator of all ATP consuming reactions is the ADP/ATP ratio and not ATP steady state per se. In the very precise ATP timed kinetic analysis, after 20 s of inhibition, the sperm ATP steady state had already decreased by more than 36%, implying an equivalent increase in ADP and drastic alteration of the ATP/ADP ratio.

The striking impact of cFEE on movement parameters is a strong incentive for searching for an impact of the peptide on energy production. The curves obtained after glycolysis or OXPHOS inhibition clearly showed no difference, depending on the sperm 3-h incubation with or without the peptide. In contrast to glycolysis, in OXPHOS, the rate of ATP production cannot suffice to determine OXPHOS bioenergetics. The same ATP production could be obtained with a high respiration rate with partial uncoupling or a low respiration rate with tight coupling. These two situations, however, would have significantly different consequences on superoxide anion production. We therefore undertook the analysis of both respiration rate and level of inner mitochondrial potential to evaluate the potential impact of cFEE. We observed similar respiration rates in sperm pre-incubated with or without cFEE. In association with similar ATP production, it logically implied that the MMP was also unaffected by the peptide. However, the very low respiration level of sperm could cast doubt on the reliability of the results. We therefore directly evaluated the impact of cFEE on MMP using fluorescent potentiometric probes and analysis by flow cytometry.

Different probes (JC1, DiOC6(3), and TMRE) have been previously used in different protocols with respect to incubation medium (composition not always explicit), probe concentrations, and additional drugs. Dissipation of the MMP, for example, was obtained with CCCP concentrations varying from 50 µM [18] to 500 µM [19,20,21] or with valinomycin from 50 nM [22] to 1 µM [23]. In our protocol, to set the MMP to a zero value, we used the joint action of the uncoupler CCCP and of the respiration inhibitor antimycin. Because antimycin blocks the respiratory chain, hence proton pumping, a lower concentration of CCCP (10µM) could be used in our analysis of sperm respiration, where this concentration was always enough to obtain maximal uncoupling, and furthermore, that concentration has been validated in several cell types [24].

For an efficient comparison of MMP values between experiments, we used the Nernst equation to obtain MMP values in mV. This approach eliminates the use of JC1, which does not follow the Nernst law requirements since it forms aggregates above a critical concentration within the mitochondrial compartment. Although TMRE and DIOC6(3) are both considered as following the Nernst law, we used TMRE for the whole set of experiments analyzing the cFEE impact because DIOC6(3) has been disputed with respect to the range of its signal [25], its possible impact on complex I [26], and potential reticulum endoplasmic staining [25].

The double population characterized by their fluorescence signal, with TMRE and DIOC6(3), was a puzzling observation that led us to use flow cytometry coupled with imaging. This new technology allowed us first to verify the specificity of the fluorescence labelling, which was indeed uniquely localized to the middle piece of spermatozoa, with DiOC6(3) as well as with TMRE. We could also verify that the population with very low fluorescence comprised only spermatozoa with normal morphology. Using this approach, we confirmed the double peak distribution of spermatozoa and the absence of cFEE impact on MMP.

Previous analyses have linked high MMP with sperm fitness either using flow cytometry coupled with cell sorting [27] with sperm movement analysis [28] or with vitality assessment [19,20,21]. Double staining of MMP and cell viability (with propidium iodide or YOPRO-1) showed that non-viable spermatozoa had low MMP, whereas viable ones contained two subpopulations differing by their MMP, which was high or at the level of the non-viable spermatozoa [19,20,21]. This observation suggests that MMP dissipation was an early alteration before loss of viability. Significantly positive correlations were observed between sperm MMP and parameters such as motility, viability, capacitation status, acrosome, and chromatin integrity, which are required for sperm function [29]. In a cohort of 91 random couples undergoing IVF, Marchetti et al. found a correlation between the fertilization rate and the percentage of high MMP spermatozoa [30]. Respiratory efficiency has also been correlated with motility [31,32] and many different OXPHOS inhibitors negatively affected sperm motility [33,34]. Evaluation of mitochondrial functions has thus been proposed to better determine the fertilizing capacity of sperm in association with the parameters routinely studied such as concentration, motility, vitality, and morphology.

With respect to these convergent data linking mitochondrial energy production and sperm fitness, the absence of cFEE impact on OXPHOS strikingly contrasts with the significant improvement induced by cFEE on the sperm movement parameters and percentage of hyperactivated spermatozoa. cFEE does not modify the total percentage of motile sperm, suggesting that it is efficient only on sperm with viable mitochondria. However, none of the OXPHOS parameters (rate of respiration or ATP synthesis, MMP) was significantly modified by cFEE, suggesting that it mostly modifies ATP consumption.

As a corollary, the absence of significant impact on OXPHOS of the peptide is reassuring for its use in human IVF. Indeed, mitochondria are not only the main source of ATP, but also the source of reactive oxygen species [35], notably via the formation of superoxide in the electron transport chain, although NADPH oxidase may also be an additional source [36,37]. Sperm capacitation, hyperactivation, acrosome reaction, and sperm oocyte interaction are linked to low levels of reactive oxygen species [38] whereas increased levels have been associated with sperm lipoperoxydation, decreased motility, DNA fragmentation, and increased apoptosis [39].

The observed cFEE-induced improvement in sperm motility, not being due to an increase in ATP production, was then likely related to a better utilization of the produced energy. Movement being produced by molecular motors, this conclusion corresponds to a gear shift. The cFEE peptide binds to the sperm membrane. Its action must therefore, most probably, use signaling pathways. Among them, cAMP or calcium pathways are central in the regulation of sperm motility [40].

## 4. Materials and Methods

### 4.1. Human Sperm

Human sperm were donated by patients undergoing an assisted reproductive technology (ART) program for in vitro fertilization (IVF) or ICSI in the assisted reproductive laboratory of Cochin’s Hospital (Paris, France) after giving informed consent. Spermatozoa were collected from excess fresh sperm derived from IVF attempts and were used on the day of collection. The GERMETHEQUE Biobank site of PARIS-COCHIN (BB-0033-00081) provided 38 samples of sperm. GERMETHEQUE obtained consent from each patient to use their samples (CPP 2.15.27). The GERMETHEQUE pilotage committee approved the design of this study under the number 20160407-01. The Biobank has a declaration DC-2014-2202 and an authorization AC-2015-2350.

### 4.2. Sperm Preparation

Semen from fertile donors was collected after three days of sexual abstinence. Sperm samples were kept at 37 °C until complete liquefaction. Motile spermatozoa were selected by a two-step 90/45% PureSperm^®^ gradient (Nidacon International, Gothenburg, Sweden) (300 g for 20 min). After washing with 2 mL of Ferticult™ medium (600 g for 5 min), they were resuspended in the same medium. Semen analysis was performed according to World Health Organization criteria [41]. The sperm were therefore kept under capacitating conditions for 3 h before use. Sperm concentration was adapted to the concentration required for each analysis. Sperm hyperactivation criteria were those of Mortimer and Swan [15].

### 4.3. cFEE Peptide Synthesis

The sequence of the disintegrin domain of human ADAM2 is CLFMSKERMCRPSFEECDLPEYCNGSSASC (accession no. CAA67753). Its binding site is composed of the three amino acid sequence FEE. The so called FEE peptide (and its biotinylated form) was synthesized by Neosystem (Strasbourg, France). Its formula, CSFEEC, contained the FEE tripeptide and was cyclized by the adjunction of a cysteine at both ends. The peptide was purified by high-pressure liquid chromatography to >95% purity. The scrambled cyclic peptide CFESEC (and its biotinylated form) was produced in a similar way.

### 4.4. Human Sperm Immunofluorescence

Freshly recovered human semen samples were divided into two groups: untreated, for the non-capacitated condition, and selected on Percoll gradient for both capacitated and acrosome reacted conditions. Up to 8 × 10^6^ of spermatozoon were used for each condition. At least three different donors were analyzed for all three conditions. Selected human semen samples were centrifuged at 600× *g* for 7 min and washed twice in PBS. Non-capacitated spermatozoa were then fixed in 2% paraformaldehyde (PFA) for 30 min at RT, washed in PBS, and stored at +4 °C. The remaining selected semen sample was centrifuged at 600× *g* for 7 min. The pellet was then resuspended in Ferticult 3% BSA and incubated for capacitation under mineral oil for 90 min at 37 °C in 5% CO_2_ in a humidified atmosphere. Half of the capacitated spermatozoa were incubated with 20 µM of calcium ionophore A23187 (Sigma) in Ferticult 3% BSA for 30 min at 37 °C. Both capacitated and acrosome reacted spermatozoa were centrifuged at 600× *g* for 7 min and washed three times in PBS 3% BSA before fixation in 2% PFA for 30 min at RT. After washes in PBS 3% BSA, spermatozoa were processed for immunofluorescence.

Spermatozoa were incubated with 100 µM biotinylated cFEE (NeoMPS) in Ferticult 3% BSA for 1 h at 37 °C. After three washes in PBS 3% BSA, spermatozoa were incubated with A594-conjugated streptavidin and FITC-conjugated PSA (Sigma, St. Louis, MO, USA) in Ferticult 3% BSA for 45 min at 37 °C. Three final washes in PBS 3% BSA were performed prior to mounting in Fluoromount G containing DAPI. To stain acrosome-reacted spermatozoon, a rabbit anti-CD46 antibody (Santa Cruz Biotechnology, Dallas, TX, USA) and a goat anti-rabbit conjugated to A633 (Life Technologies, Thermo Fisher Scientific, Chicago, IL, USA) were used, along with the biotinylated peptide incubated, along with the A594-streptavidine. Image acquisition was performed using a spinning disk SP5 microscope. The counts were carried out using a Nikon Eclipse E600 fluorescence microscope. At least 300 spermatozoa were counted for each donor and each condition.

### 4.5. Acrosome Reaction Assessment

Spontaneous acrosome reaction was assessed after incubation of spermatozoa with 100 µM cFEE or scramble peptide for 18 h at RT. Sperm from eight patients were tested. After capacitation, each selection was divided into two groups that were incubated with either 100 µM cFEE or scramble peptide for 1 h at 37 °C. Then, a detection of acrosome reacted sperm was achieved using an anti-CD46 antibody conjugated to FITC (Ancell, Bayport, NY, USA) that stains the inner acrosomal membrane. Sperm were incubated with 10 µg/mL antibody for 1 h at 37 °C in the dark. After two washes in PBS BSA (1%) and 5 min centrifugation at 600× *g*, the sperm suspension was fixed using 2% PFA for 1 h at RT. Then, all sperm were labeled using propidium iodide (Invitrogen, Illkirch, France) to stain their DNA and render them easier to detect by flow cytometry. Finally, the proportion of acrosome reacted sperm was measured on each sample by flow cytometry using a BD FACS Canto II (BD Biosciences, San Jose, CA, USA). Similarly, assessment of the percentage of induced acrosome reaction was conducting after incubation with cFEE or scramble peptide. The acrosome reaction was induced by incubating the sperm with 20 µM of calcium ionophore A23187 (Sigma Aldrich, Saint Quentin Fallavier, France) for 30 min at 37 °C.

### 4.6. Analysis of Sperm Vitality, Motility and Sperm Movement Parameters

Sperm vitality was assessed after selection on the Percoll gradient. Vitality was quantified by flow cytometry analysis of 10,000 events using a FACS Canto II cytometer from Becton Dickinson, and propidium iodide. Sperm were gated through their size and granularity. To potentiate the analysis of peptide toxicity on sperm vitality, a concentration of 200 µM was tested for this experiment.

Sperm concentration and motility (total and progressive) were evaluated at 37 °C for computer-assisted sperm analysis (CASA) (Hamilton Thorne, Beverly, MA, USA). Samples were placed in a 20 µm deep chamber. The configuration parameters were: teacher ratio 60 Hz, intensity of illumination 1.89, refresh time 0.5 s, and threshold of 16 points minimum benchmarks. Motility was validated for a minimum of 150 sperm analyses. The analyzed sperm movement parameters were: the curvilinear velocity (VCL curvilinear velocity), the speed of progress or smoothed value of the VCL (VAP average path velocity), the rate of progression of the cell (VP or VSL for straight line velocity), and the lateral displacement amplitude sperm head (ALH amplitude of lateral head displacement). The criteria used to define hyperactivated sperm were: ALH > 6 µm, VCL > 180μm/s, LIN (VSL/VCL) ≤ 45%, and WOB (VAP/VCL) < 50% [15].

### 4.7. DNA Fragmentation Analysis

DNA fragmentation was assessed according to the modified method of Mitchell et al. [42]. Indeed, we showed that when the sperm concentration in the aliquot was lower than 5 × 10^6^/mL, the results were poorly reproducible. Therefore, we selected sperm samples that contained at least 10^7^ spermatozoa. For this reason, several normal sperm were selected and pooled. Three samples of 10^7^ spermatozoa were taken from this pool and treated for 3 h at 37 °C with either 100 µM cFEE, 100 µM scramble, or medium alone (control). These samples were then washed with Ferticult medium and centrifuged for 5 min at 600× *g*. A LIVE/DEAD incubation (Life Technologies, Villebon-sur-Yvette, France) was performed according to the manufacturer’s recommendations. Sperm were then washed, and sperm DNA decondensation was induced using 2 mM DTT for 45 min at room temperature, prior to fixation in PFA (4%). Finally, a TUNEL assay was run according to the technique already described [42]. Flow cytometry analysis allowed for the determination of the degree of DNA integrity of each sample.

### 4.8. Glycolysis and OXPHOS Inhibitors

OXPHOS inhibitors targeted either the respiratory chain with antimycin 1µM (complex III) or cyanide 2mM (complex IV), or the ATP synthase (complex V) with 8 µM oligomycin (OM). The protonophore carbonyl cyanide m-chlorophenyl hydrazone (CCCP) was used as an uncoupler. To set the mitochondrial membrane to zero value, two procedures were used: the joint action of CCCP 10 µM and of the respiration inhibitor antimycin, or alternatively, the addition of the ionophore valinomycin (5 µM) with which the free/uncontrolled permeation to potassium is expected to collapse the mitochondrial membrane potential.

Glycolysis inhibitors were 20 mM 2-deoxyglucose, which blocks glycolysis at the level of the first phosphorylation step, and 20 µM iodoacetate, which blocks glycolysis at the level of the glyceraldehyde 3-phosphate dehydrogenase (GAPDH) (OXPHOS and glycolysis inhibitors were purchased from Sigma, St. Louis, MO, USA).

### 4.9. Sperm ATP Content

Sperm ATP content was analyzed in sperm suspended in Ferticult at 6.10^6^/mL or higher. A total of 15 µL aliquots of sperm suspension were taken twice before the addition of inhibitors, and then 20, 40, and 60 s after the addition of either OXPHOS or glycolysis inhibitors. To prevent cellular reactions, potentially producing and/or using ATP, the 15 µL aliquots were immediately transferred into 85 µL of lysis buffer (HS kit II Roche), vigorously mixed, and stored at −80 °C until the ATP assay. ATP concentration was measured after dilution of each aliquot in three volumes of water using the ATP Bioluminescence Assay Kit (HS kit II Roche) on a plate reader (BMG) as previously described [43].

### 4.10. Oxygen Consumption Rate

Oxygen consumption of 10 to 20 million of spermatozoa per mL was determined at 37 °C by high-resolution respirometry (Oroboros Oxygraph-2K, Innsbruck, Austria) as described [44]. Basal respiration was measured for 15 min, followed by the addition of 1 µM oligomycin, to assess coupling. Then, 1.25 µM carbonyl cyanide-*P*-trifluoromethoxy-phenylhydrazone (CCCP) was included by successive additions to assess maximal respiration. The final addition of 1 mM KCN allowed us to assess the non-respiratory oxygen consumption. This non-respiratory oxygen consumption was subtracted from all the values to yield the mitochondrial respiratory rate in the different conditions (basal, oligomycin, maximal). These values were then used to calculate the respiratory control as the ratio basal/oligomycin and the respiratory reserve as the difference between maximal and basal respiration.

### 4.11. Evaluation of the Inner Mitochondrial Membrane Potential by Standard Flow Cytometry and with Imager

Fluorescent lipophilic cations, whose concentration in the mitochondrial compartment is driven by MMP, serve as probes for MMP measurement. The most commonly used are 5,5′,6,6′-tetrachloro-1,1′,3,3′-tetraethylbenzimidazolyl-carbocyanine iodide (JC-1), 3,3′-dihexyloxacarbocyanine iodide [DiOC6(3)] and tetramethylrhodamine ethyl ester (TMRE). All of them have been used to measure sperm potential [19,20,21]; JC-1 is set apart because forming aggregates within the mitochondria, it cannot be considered as following the Nernst equation used for formal potential measurement.

Potentiometric fluorescent probes were used at 200 nM for JC-1, 100 nM for TMRE, and 20 nM for DiOC6(3). Stock solutions were prepared at 100X concentration in dimethylsulfoxide (DMSO) and stored in small aliquots at −20 °C. Dilution to final concentration was performed immediately before use in 0.5 mL Ferticult medium containing 5.10^5^ spermatozoa. After incubation at 37 °C in the dark for 30 min [19,20,21], the sperm suspension was read in triplicate on the BD TM Accuri cytofluorometer using CFlow sample software for data acquisition. Spermatozoa were gated based on forward and side scatters and a minimum of 10,000 spermatozoa were analyzed. DiOC6(3) fluorescence was read through the FL1 channel (530 ± 15 nm band pass filter); TMRE fluorescence through the FL2 channel (585 ± 20 nm band pass filter) and FL3 channel (>670 nm band pass filter); and JC-1 fluorescence was read through the three channels, with FL2 and FL3 channels reading the red signal from JC-1 aggregates.

Control experiments comprised the dissipation of the MMP, either with 5 µM valinomycin or the association of 2.5 µg/mL antimycin + 10 µM CCCP, and the inhibition of complex V with 1 µM oligomycin.

Since the FCM analysis showed two peaks of fluorescence, we tried to determine their nature using ImageStream^®^X Mark II (AMNIS Corp., Seattle, WA, USA), which combines flow cytometry with microscopy. Probe concentration and incubation were similar to those used in standard cytometry except for the sperm concentration, which was set at 2.10^6^/mL. Spermatozoa were selected by analysis of the bright field images, keeping only the field in which a single object with a surface above 75 pixels could be seen. Fluorescent signals were read in channels similar to those used in standard flow cytometry.

Histograms of sperm fluorescence intensity were built using IDEAS software analysis (AMNIS Corp., Seattle, WA, USA), allowing us to define the population of “non-fluorescent sperm” and to calculate the mean fluorescence intensity in either total sperm population or after subtraction of the “non-fluorescent sperm”.

### 4.12. Statistical Analysis

Quantitative variables were described by their number, average, and standard deviation or standard error mean. Comparison of two groups was performed using the non-parametric Wilcoxon–Mann–Whitney test unless a normal distribution of the data allowed using a Student *t* test. A two-way ANOVA and Sidak’s multiple comparisons test were used for analysis of cFEE localization. Analysis of the cFEE peptide impact was done using the paired test in order to take into account potential bias related to differences between individual sperm donors. Comparison of more than two groups used a Kruskal–Wallis ANOVA on ranks followed by a multiple comparison procedure with the Tukey test or, when data distribution was compatible, with ANOVA followed by a multiple comparison procedure with the Holme–Sidak test. Comparison of percentages was conducted using a Chi-2 test (χ2) or Fisher exact test. Statistical significance was set at a *p* value below 0.05.

## Figures and Tables

**Table 1:** cFEE increases sperm movement parameters.

Parameters	Scramble (*n* = 38) Mean ± SD	cFEE (*n* = 38) Mean ± SD	*p* Wilcoxon
VAP	80.5 ± 16.8	85.9 ± 13.8	**0.008**
VSL	66.8 ± 16.7	71.2 ± 15.5	0.048
VCL	136.5 ± 26.9	147.1 ± 26.7	**<0.0001**
ALH	6.1 ± 1.3	6.6 ± 1.3	**0.002**
BCF	33.0 ± 3.2	33.2 ± 3.2	NS
STR	84.4 ± 11.9	84.4 ± 11.2	NS
LIN	50.5 ± 11.3	49.8 ± 12.5	NS
Hyperactivation	10.5 ± 9.3	13.6 ± 11.2	**0.009**
Total motility	74.2 ± 14.6	76.9 ± 12.2	NS

Average values of the parameters of sperm movement assessed by CASA in the presence of 100 µM of cFEE peptide or control scramble peptide (90 min incubation). Statistical tests were nonparametric tests for paired values (Wilcoxon test). APV = average path velocity, SLV = straight line velocity, CLV = curvilinear velocity, ALH = amplitude of lateral head displacement; BCF = beat/cross frequency; STR = straightness (=SLV/APV); LIN = linearity (=SLV/CLV).

**Table 2:** cFEE does not affect sperm vitality.

Concentration (µM)	Control (*n* = 8)	cFEE (*n* = 8)	Scramble (*n* = 8)	*p*
10	57.8 ± 22.2	60.1 ± 21.5	54.8 ± 25.8	NS
50	52.4 ± 23.0	54.2 ± 26.8	58.2 ± 19.8	NS
100	51.9 ± 19.4	61.1 ± 16.9	58.2 ± 20.5	NS
200	63.2 ± 15.8	65.4 ± 17.6	62.2 ± 16.4	NS

Means and standard deviations for different sperm and molarities of cFEE and scramble peptides compared to vitalities observed with the same sperm in the presence of equivalent dilutions made with the culture medium after 90 min of incubation. The differences were not significant (NS) (Wilcoxon test).

## Supplementary Materials

The Supplementary Materials are available online at https://www.mdpi.com/article/10.3390/ijms222011263/s1.

## Abbreviations

DOAJ	Directory of open access journals
TLA	Three letter acronym
LD	Linear dichroism
CASA	Computer-assisted sperm analysis
cQDE	Cyclic peptide (Glutamine; Aspartic acid; Glutamic acid: Gln-Asp-Glu)
cFEE	Cyclic fertilin peptide (phenylalanine, glutamic acid; glutamic acid: Phe-Glu-Glu)
MMP	Mitochondrial membrane potential
OXPHOS	Oxidative phosphorylation
