# Supplementary material for: Cyclic FEE Peptide Improves Human Sperm Movement Parameters without Modification of Their Energy Metabolism
Source: Int J Mol Sci. 2021 Oct 19;22(20):11263. doi: 10.3390/ijms222011263 (PMC8539654; doi:10.3390/ijms222011263)
Supplement: Supplementary file 1 [file ijms-22-11263-s001.zip › ijms-1424284-supplementary.pdf]

Article

Cyclic FEE peptide improves human sperm movement parameters without modification of their energy metabolism.

Nathalie Le Foll [1,2], Jean-Christophe Pont [1], Audrey L'Hostis [1,2], Thomas Guilbert [1], Frédéric Bouillaud [1], Jean-Philippe Wolf [1,2,*,£] and Ahmed Ziyyat [1,2,£]

[1] Université de Paris, Institut Cochin, INSERM, CNRS, F-75014 PARIS, France.
[2] Service d'histologie, d'embryologie, Biologie de la Reproduction, AP-HP, Hôpital Cochin, F-75014 PARIS, France.
[£] These authors contributed equally to this work
* Correspondence: jean-philippe.wolf@aphp.fr, Tel.: 33 (1) 58 41 11 71

Supplementary Materials:

Supplementary Materials:

S1. Energetic requirement for sperm movement

All the sperm analyses were performed in Ferticult medium since it is the medium that was used to assess the cFEE effects on sperm movement. To analyze the metabolic pathway of ATP production we had first to determine its major composition regarding energetic pathway supply. We analyzed Ferticult composition with a Cobas 8000 (Roche Diagnostics, France) and found that it contains substrates for both glycolysis (5.8 mM glucose) and OXPHOS (0.5 mM pyruvate, 12.5 mM lactate). To evaluate the relative influence of each energetic pathway on movement, we compared the movement parameters, analyzed with CASA, of five independent samples from sperm donors untreated, treated with glycolysis inhibitor (iodoacetate) or treated with the OXPHOS inhibitor oligomycin. The impact on movement was observed immediately after addition of the inhibitors (Table S1 and Figure S1).

Table S1. Glycolysis and OXPHOS energy for sperm movement.

	**Basal**	**Glycolysis inhibition**	**OXPHOS inhibition**
VAP (µm/s)	57.8	**29.8** *	**9.3** ***
VSL (µm/s)	50.1	**23.0** *	**5.1** ***
VCL (µm/s)	96.0	**60.2** *	**21.1** **
ALH (µm)	4.5	3.6	**1.3** **
BCF (%)	31.8	33.0	**15.6** **
STR (%)	83.4	75.5 *	**22.4** **
LIN (%)	51.2	**37.5** **	**10.2** **
total motility (%)	70.2	**34.0** **	**1.8** ***
progressive motility (%)	47.0	**12.4** ***	**0.2** ***

APV =Average path velocity, VSL=Straight line velocity, VCL=Curvilinear velocity, ALH=amplitude of lateral head displacement; BCF=Beat-Cross frequency; STR=Straightness (=VSL/VAP); LIN=Linearity (=VSL/VCL); *=P<0.05; **=P<0.01; ***=P<0.001 comparing with basal conditions.

OXPHOS inhibitors induced the complete arrest of sperm movement, immediately for three samples and in less than two minutes for the two others. Glycolysis inhibitors immediately induced a significant but partial decrease of sperm movement. The movement parameters continued to decrease within the 15 min of the study (Figure S1). These results show that both energetic pathways are required for sperm movement. OXPHOS however appears predominant in our conditions of sudden inhibition.

Figure S1

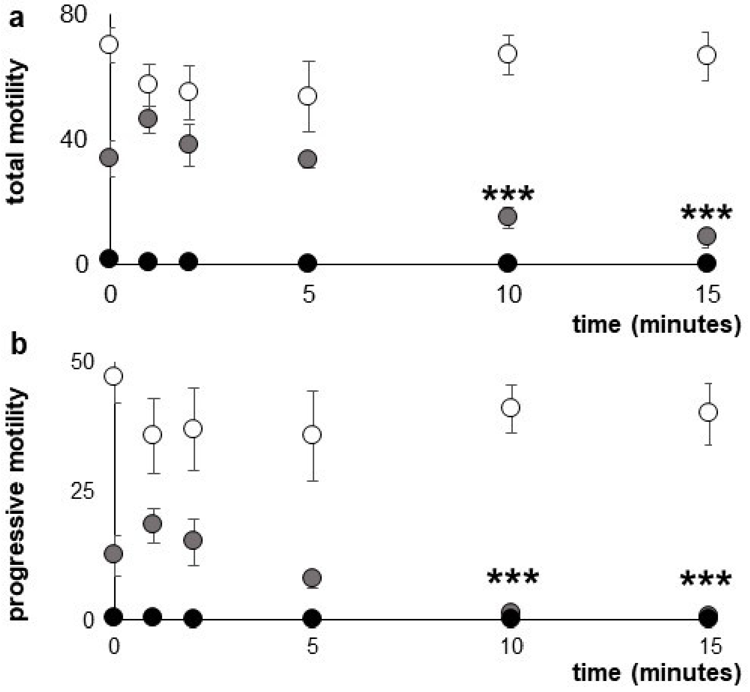

Figure S1. Energetic requirement of sperm movement: Upon OXPHOS inhibition, sperm movement essentially stopped whereas it was partially maintained upon glycolysis inhibition but progressively deteriorated. The graph shows the evolution with time of total motility (**a**) and progressive motility (**b**) taken as representative of the evolution of sperm movement; values are means ± sem from 5 independent samples in basal conditions (white circles), upon glycolysis inhibition (gray circles) or upon OXPHOS inhibition (black circles); *** $P<0.001$ when comparing with values observed at time 0 minute.

To quantify the respective part of glycolysis and OXPHOS in the ATP production, we directly measured ATP concentration before and after inhibition of each of these pathways (Figure S2). ATP concentration was expressed as picomoles/10^6 sperm, measured twice at one-minute interval, in 12 samples from 4 different sperm donors (Figure S2**a** and **b**). In the absence of inhibition, ATP concentration remained constant, reflecting the balanced equilibrium between production and consumption (Figure S2**a**). The decrease in ATP concentration was very significant for both OXPHOS or glycolysis inhibition at $P<0.001$ with ANOVA on ranks on data expressed as percentages of the initial level of ATP, and at $P<0.05$ when comparing ATP concentration before inhibition with that observed after at 20, 40 or 60 seconds, using Dunn's method for pairwise multiple comparisons. Its initial decrease upon inhibition therefore reflects the rate of the abolished production. The steeper decrease of ATP concentration upon OXPHOS inhibition than upon glycolysis inhibition confirmed the predominant role of OXPHOS in ATP supply in these conditions (Figure S2**b**). Exponential regression curve fitted with an excellent correlation

coefficient in both cases (above 0.95). We therefore used the exponential equation to calculate the ATP decrease during the first five seconds and obtained a rate of 360 picomoles per minute in the case of OXPHOS inhibition and 271 picomoles per minute in the case of glycolysis inhibition. In sperm, OXPHOS thus generates roughly one third more ATP than glycolysis.

Figure S2

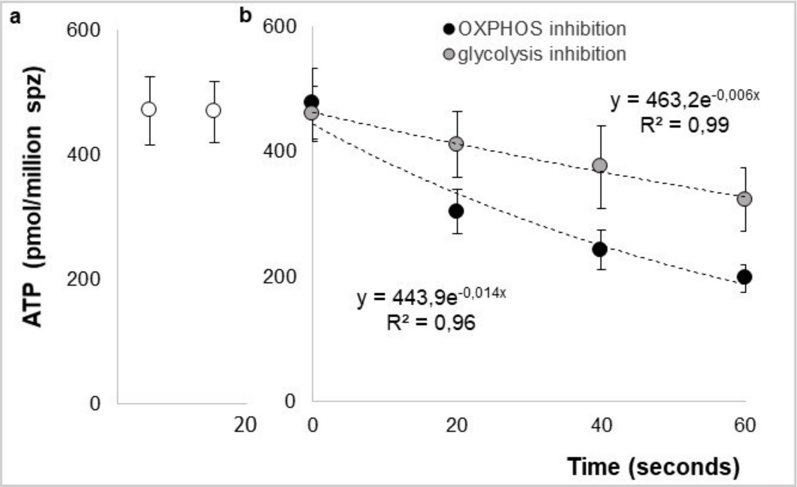

Figure S2. **Sperm ATP production. a.** ATP concentration is stable in untreated sperm; ATP concentration expressed as picomoles/10^6 sperm, measured twice at a one-minute interval, in 12 samples from 4 different sperm donors; values are mean ± SD. **b.** Both glycolysis and OXPHOS inhibition induced a significant decrease of ATP concentration; kinetics of the ATP concentration of 12 samples after glycolysis inhibitors (gray circles) or OXPHOS inhibition (black circles); dotted lines = exponential regression curve whose equation and correlation coefficient are in the upper part of the graph for glycolysis inhibition and in the lower part for OXPHOS inhibition.
